# Vascular-targeted photodynamic therapy with TOOKAD^®^ Soluble in localized prostate cancer: standardization of the procedure

**DOI:** 10.1007/s00345-015-1535-2

**Published:** 2015-03-19

**Authors:** Abdel-Rahmene Azzouzi, Souhil Lebdai, Fawzi Benzaghou, Christian Stief

**Affiliations:** 1 Urology Department, CHU Angers, 4 rue Larrey, 49933 Angers Cedex 9, France; 2 STEBA Biotech, Paris, France; 3 Department of Urology, Ludwig-Maximilians-University, Munich, Germany

**Keywords:** Localized prostate cancer, Focal therapy, WST11 TOOKAD^®^ Soluble, Vascular-targeted photodynamic therapy

## Abstract

**Introduction:**

Vascular-targeted photodynamic therapy with TOOKAD^®^ Soluble is an innovative focal therapy procedure assessed in localized prostate cancer treatment.

**Materials and methods:**

This mini-invasive technique destroys targeted tissues using a photosensitizer [TOOKAD^®^ Soluble (WST11), STEBA Biotech] activated by laser light in the presence of oxygen. Its application for prostate cancer requires intravenous infusion of TOOKAD^®^ Soluble and the illumination of the targeted area by transperineal optical fibers inserted under trans-rectal ultrasound guidance under general anesthesia.

**Conclusion:**

Based on the experience gained through hundreds of procedures, we describe here the standardized technique of vascular-targeted photodynamic therapy with TOOKAD^®^ Soluble defined during the phase II and III trials.

## Introduction

Vascular-targeted photodynamic therapy (VTP) with TOOKAD^®^ Soluble is a focal therapy procedure currently assessed in localized prostate cancer treatment [1–3]. VTP destroys targeted tissues using a photosensitizer [TOOKAD^®^ Soluble (WST11), STEBA Biotech] in association with a low power near-infrared laser light in the presence of oxygen. The photosensitizer absorbs light and transfers energy to oxygen molecules creating reactive oxygen species inducing local vascular occlusion resulting in cell destruction [4]. It is intravenously infused and circulates systemically while only the targeted area of the prostate is illuminated [5]. It induces irreversible damage to cell membranes and small arterioles [6] and blocks blood and nutriment supply to tumors by extensive effects on tumoral vasculature by taking advantage of its sensitivity to stress [7]. Damage to vascular endothelium is quickly followed by a cascade of events, including thrombosis, blood stasis, and vessel occlusion, leading to tumor necrosis [8–10].

TOOKAD^®^ Soluble VTP procedure is indicated in patient suffering from localized prostate considered eligible for focal treatment. Optimal results are obtained for prostate volume between 25 and 70 cc.

The absolute contra-indications are as follows: a prior prostate surgery, an acute urinary retention in the last 6 months, and history of urethral stricture disease. This procedure was first performed in 2004 by John Trachtenberg in the Princess Margaret Cancer Center in Toronto on a series of patients presenting recurrent prostate cancer following radiation failure. It was then imported in Europe by Mark Emberton at the University College Hospital, London.

The aim of this article was to report the technical aspects of the TOOKAD^®^ Soluble VTP procedure and how it has been standardized during the phase II and III trials in Europe, North America and Latin America.

## Pre-operative preparation of the patient

An alpha-blocker was advised a week before the procedure for a period of 1 month in order to ease the lower urinary tract symptoms (LUTS) due to prostate swelling during the immediate postoperative period (2–3 weeks).

A rectal preparation was performed the day before surgery.

### The treatment guidance by imagery

**Fig. 1:**
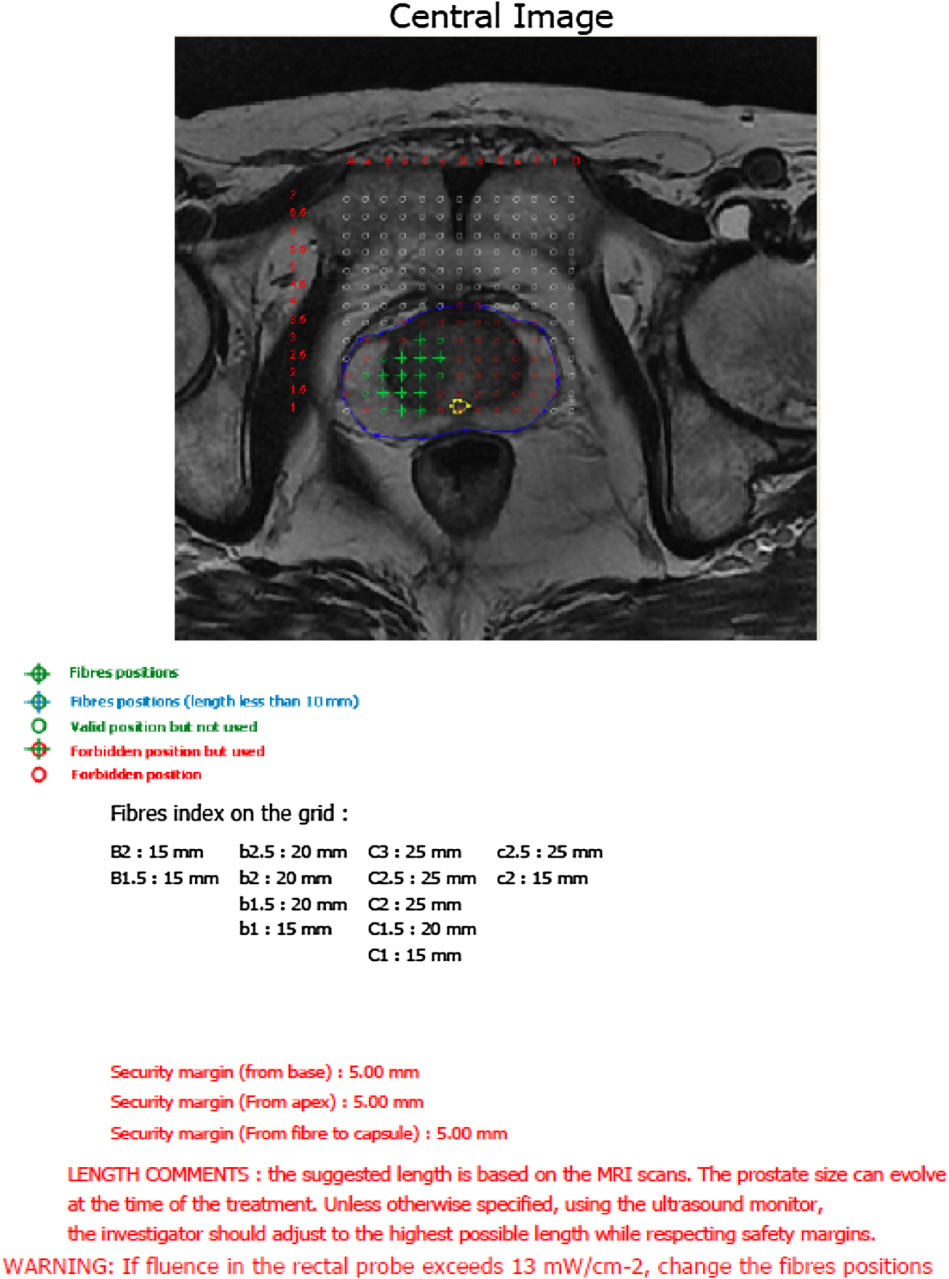

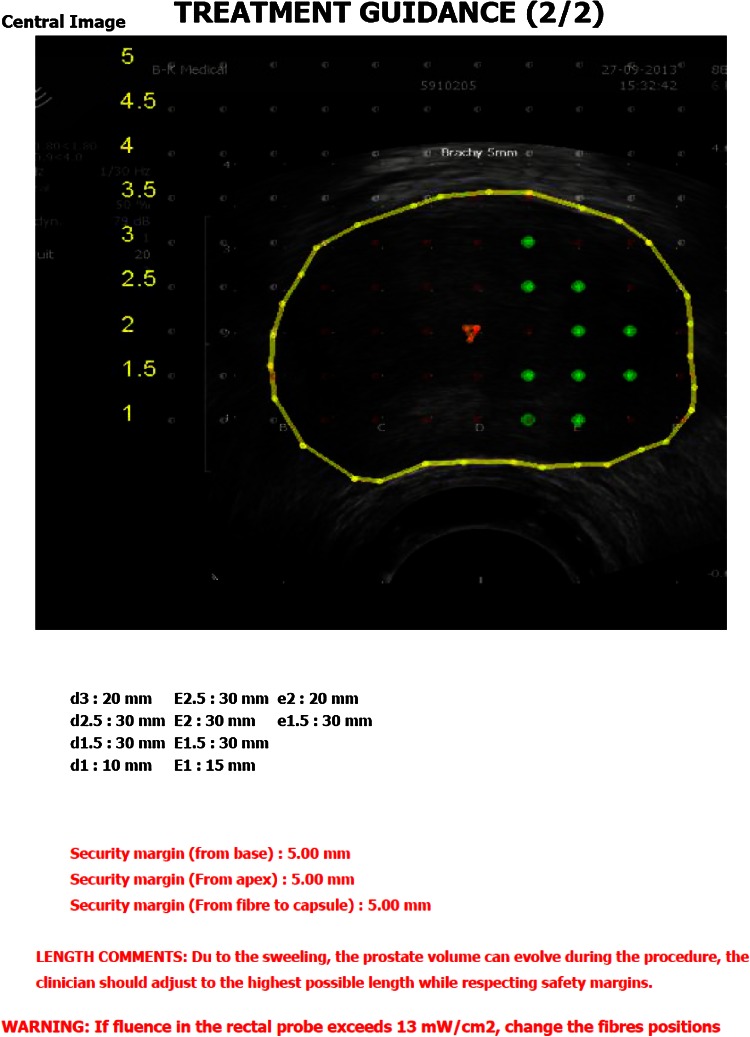
TOOGUIDE^®^ treatment guidance. The position of the fibers is determined on either prostatic MRI/or prostatic ultrasound

The treatment guidance by imagery (either by MRI or by ultrasound) permitted to define the target volume to treat, the safety margin regarding the urethra, posterior, lateral capsula and rectal wall. The integrity of these anatomical structures had to be strictly respected during the procedure. It was performed with a software called TOOGUIDE^®^ (Stebabiotech) which optimizes the parameters of treatment: number of optical fibers, accurate position of the fibers into the prostate and the length of the light diffusing of each optical fiber (Steba-Biotech) [11] (Fig. 1).

## Installation

General anesthesia was mandatory. Peridural or local anesthesias were not indicated, and curarization was recommended because of the absolute necessity of a complete immobility of the patient during the whole procedure in order to keep the safety and the efficacy of the treatment in optimal conditions. Indeed, any movement of the patient during the procedure should lead to a complete reinstallation of all transperineal fiber insertion catheters (FIC). The Anesthesiologist was informed of this requirement before the procedure, and a zero movement rule was mandatory.

The patient was placed in lithotomy position at the edge of the operating table with enough hip flexion in order to expose the perineum without being hindered by the pubis. As in brachytherapy, the legs had to be spread to avoid a conflict between the pubic arch and the insertion of the FIC.

**Fig. 2:**
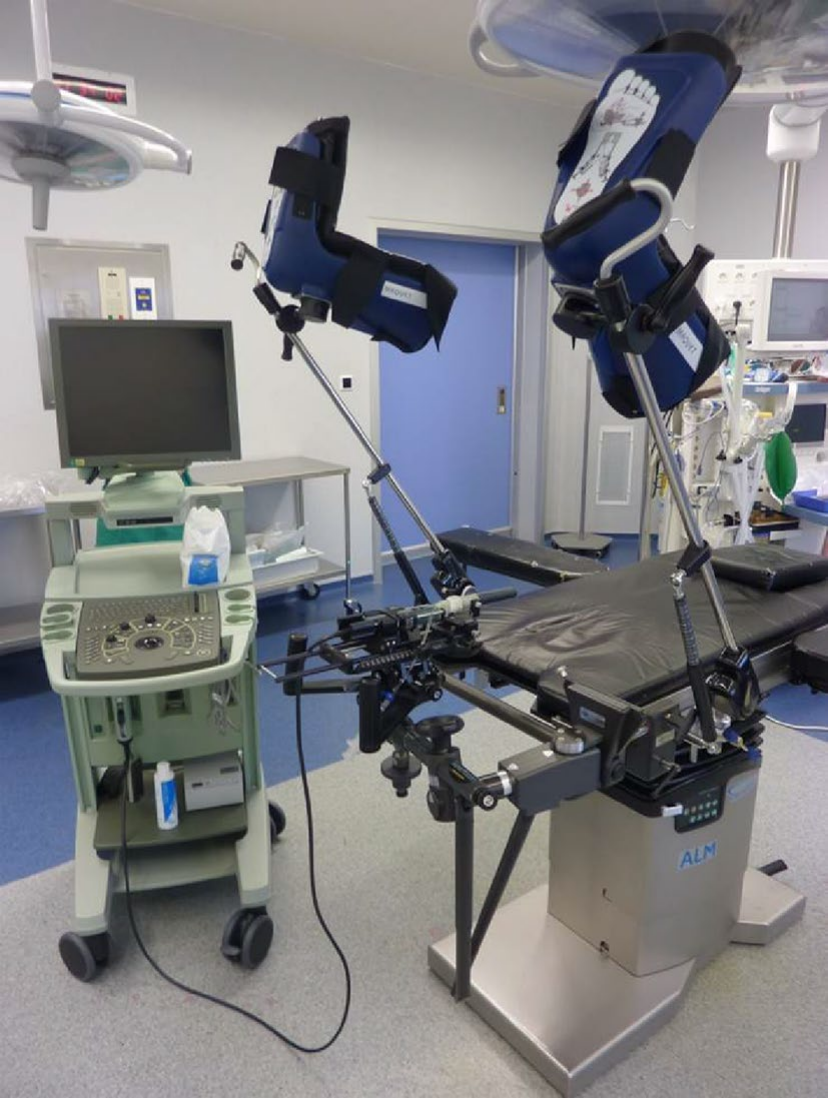
Installation of the operating table, the ultrasound device and the brachystepper

**Fig. 3:**
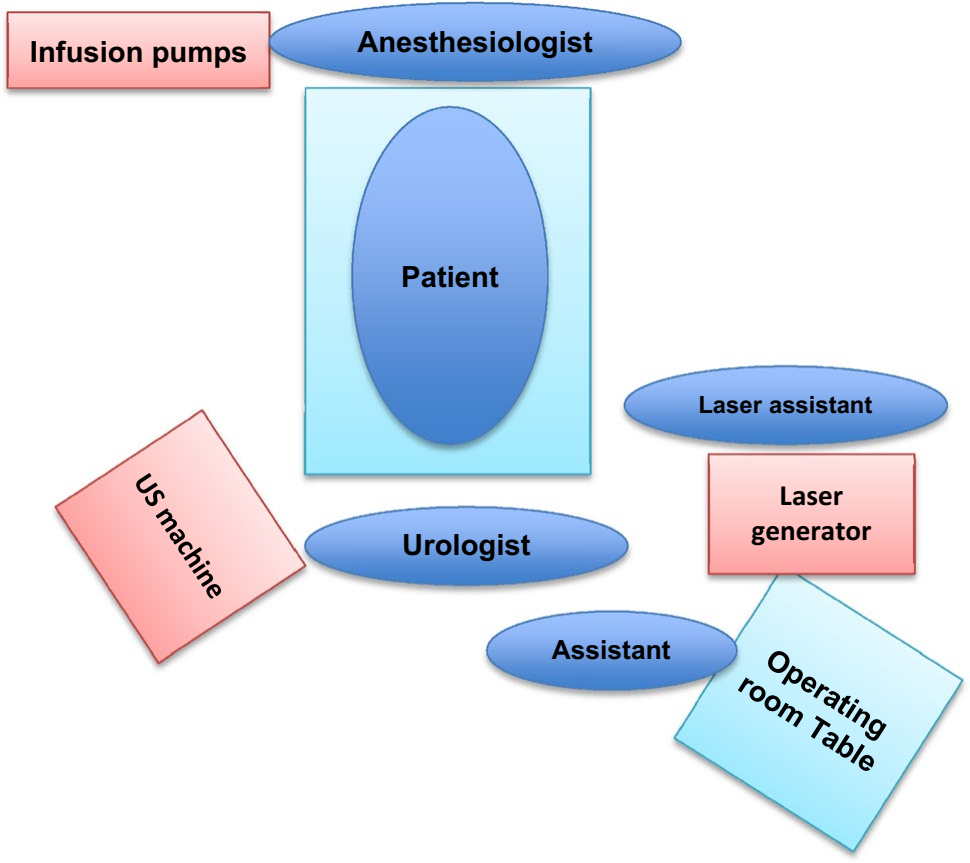
The operating room organization. The position of the material (*right* or *left*) depends on the habits of the urologist

Conventionally, the ultrasound machine was on the left of the surgeon, the nurse, her table and the laser, on the right (Figs. 2, 3).

**Fig. 4:**
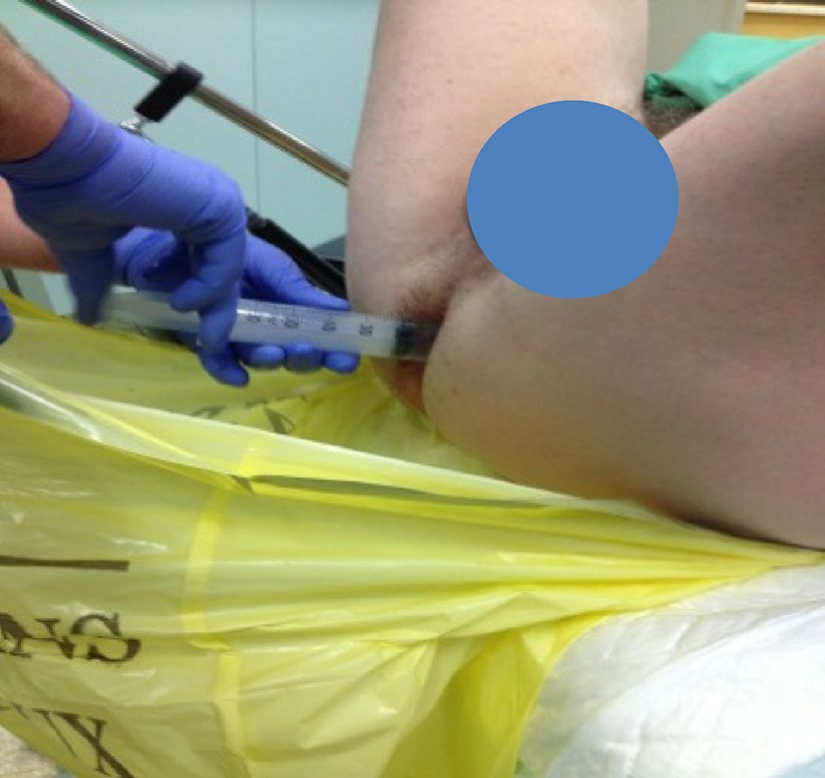
Cleaning the rectum

The prostate and the adjacent structures were visualized by the biplane trans-rectal ultrasound probe attached to a standard brachytherapy stepper (Civco, USA) with an attached transperineal prostate template (i.e. Mic Radio Nucelar Instruments Inc 0308-02-13 B&K or compatible). Biplane vision was mandatory in the choice of the TRUS scan machine. Before the insertion of the TRUS probe, it was capital to make the rectum perfectly clean with no residual feces. A feeding syringe with saline solution can be used. This point was capital since the image had to remain optimal during the whole procedure especially because there is no opportunity to correct it once started (Fig. 4).

**Fig. 5:**
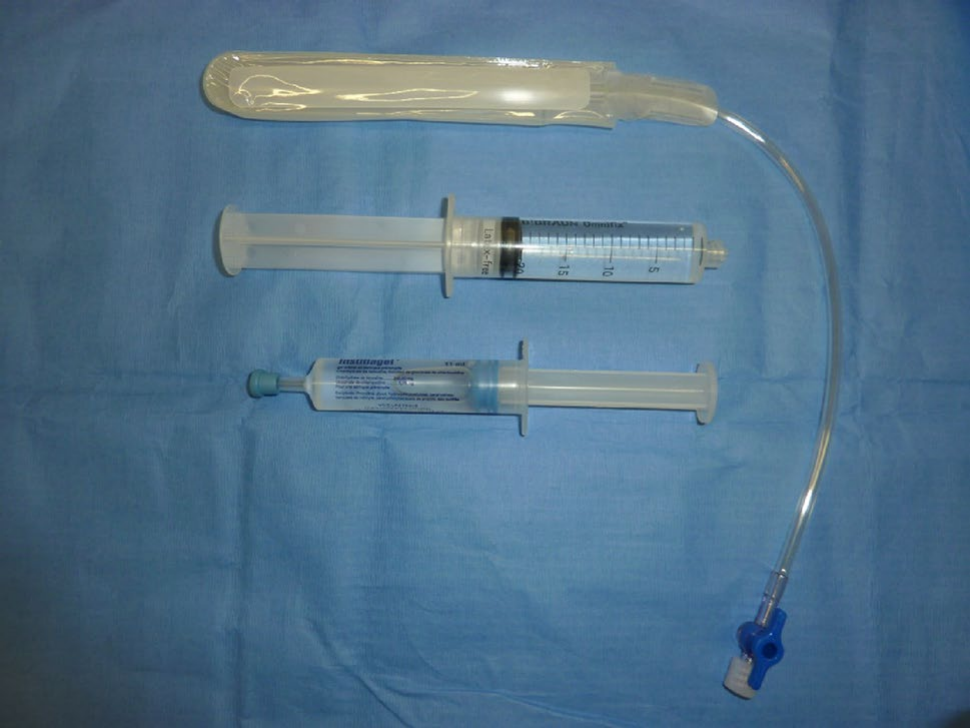
The balloon, the saline syringe and the urethral gel

The probe was covered by a protective endo-cavity balloon previously filled with urethral gel. The urethral gel was more appropriate than ultrasound gel because it contains fewer bubbles and facilitates the introduction of the probe in the balloon (Fig. 5). Thus, the quality of the picture improved in order to reach an MRI-like definition. The probe preparation should be performed with the probe on the US machine fixed into the stabilizer of the brachystepper in order to avoid any damage to the probe and to allow the surgeon to perform this step without any external help. The balloon was strongly fixed by a rubber band to avoid TRUS probe movements inside the balloon. Otherwise, the balloon may stay stuck in the rectum allowing the probe to move in and out the balloon.

**Fig. 6:**
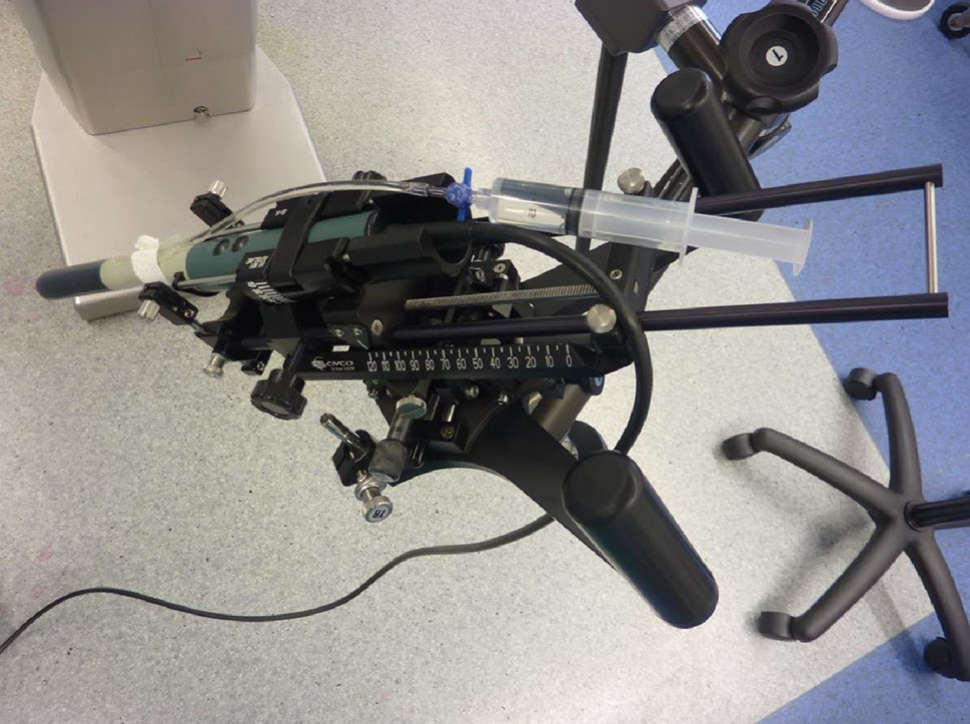
The ultrasound probe, the balloon and the brachystepper device

Before introducing the TRUS probe in the rectum, the brachystepper had to be set on a neutral position to allow movements in all directions. Pointing the stepper toward the floor while inflating the balloon removed spontaneously the residual bubbles (Fig. 6). Ten to fifteen cc of saline was injected into the balloon using a 20-cc luer-lock syringe in order to optimize the passage of the ultrasounds through the acoustic window without compressing the posterior part of the prostate. This detail was important since the compression of the prostatic tissue and particularly the peripheral zone would modify the shape of the targeted volume and might modify the blood flow in the prostate and compromise the efficacy of the technique. Furthermore, by compressing the prostate, the distance FIC–FIC or FIC–capsula would be artificially reduced during the procedure. Eventually, inflating the balloon allowed to adjust the position of the prostate on the screen.

When the probe was introduced into the rectum, it was advised to remove it two or three times in order to empty the remaining saline trapped into the rectum after its cleaning. Once the TRUS probe was in position, an optimal picture was sought by adjusting: the frequency to 5–6 MHz, the width by focusing the ultrasounds on the prostate, the depth to increase the size of the prostate at its maximum on the screen, and the resolution of the image. Once an optimal image was obtained, the stepper device was locked, and only longitudinal (superior–inferior) movements were permitted thereafter.

Using the TOOGUIDE TRUS^®^ software (Steba-Biotech), the US images of the prostate were acquired by steps of 5 mm from the basis to the apex by mobilization of the stepper. The pictures were saved on the ultrasound machine and transferred to the software. The Urologist had to delineate the prostate limits and to launch the software in order to produce the treatment guidance within 2 min (Fig. 1).

The patient’s skin was scrubbed with an antiseptic such as povidone iodine or chlorhexidine or equivalent; then, the patient was draped with two leg drapes, and then a sterile transparent plastic drape covered the brachystepper and the probe (eventually two medium-sized drapes delimitating the surgical field including the penis and the perineum). These two drapes were fixed to the transparent drape to completely close the sterile operative area. The keyboard of the ultrasound machine was also covered with a transparent sterile drape to allow the urologist to independently manage the US measurements.

The bladder was drained through a Foley catheter placed in the urethra, and the balloon was inflated with 10 cc saline. The catheter had to be pushed far in the bladder and fixed to the glans with a sticky tape in order to avoid blowing up the balloon during the procedure. The catheter was clamped with a spigot during the whole procedure in order to keep the bladder full which will improve the visualization of the limits of the prostate by ultrasound.

The Anesthetist should avoid excess fluid supply as a full bladder could trigger movements and even waken the patient, as well as it could alter the shape of the prostate by compressing its base.

The patient was integrally protected from light exposure. The only exposed zone was the perineum.

## Procedure

**Fig. 7:**
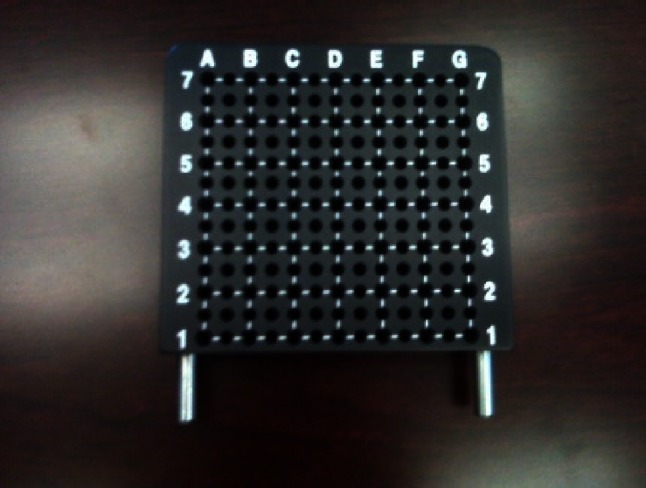
Reusable transperineal prostate template

**Fig. 8:**
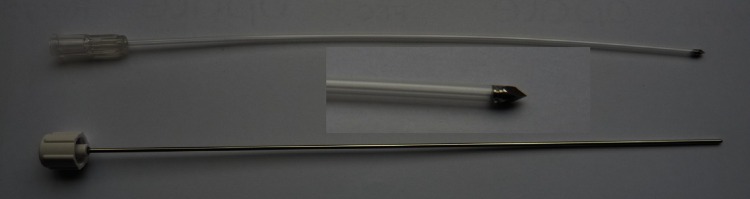
Fiber insertion catheter

Once the installation was achieved, transparent hollow FICs were positioned into the prostate transperineally through the template (Figs. 7, 8), using the TRUS scan system in accordance with the treatment guidance provided by the software TOOGUIDE^®^. The virtual template grid on the screen increased the accuracy of the FIC placement. Using FICs with metallic tips facilitated their penetration through the skin and the prostate. The best way to introduce the FIC was to do it swiftly. A slow movement would make the skin more difficult to pass through and displace the prostate leading to a wrong position of the FIC into the targeted area. Conventionally, the FICs were inserted from anterior to posterior and from right to left. By using this method, the visualization of the posterior FICs during the introduction was not disturbed by the artifacts due to the FICs already in place. The right to left convention was very helpful to label each FIC and its corresponding optical fiber in a right and reproducible order.

**Fig. 9:**
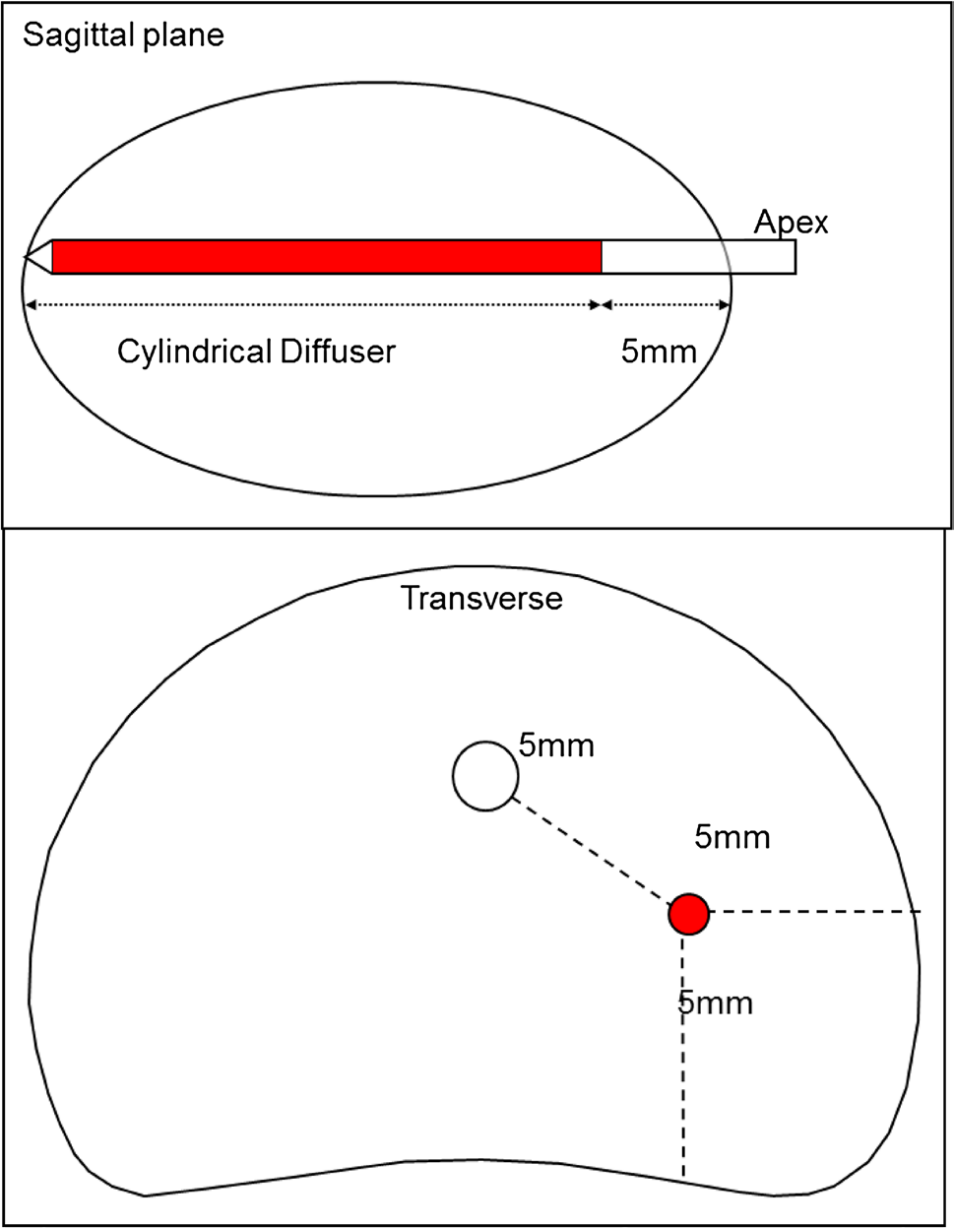
Safety margin for the optical fiber positions

Once all the FICs were in place, the accurate measurement of each corresponding optical fiber was performed. The positions of the optical fibers into the FICs must respect a safety margin of 5 mm to the urethra, the rectal wall, the sphincter and the capsula (Fig. 9). The diffusing lengths of the optical fibers (10–50 mm) were chosen according to the sagittal length of the prostate minus 5-mm safety distance from the apex. According to each measurement, the corresponding diffusing optical fibers were inserted into the FIC. Before the insertion, these optical fibers had to be calibrated by inserting their illuminating diffusor into the integrating sphere in the laser generator, in order to adjust the power with the planned values (within ±5 mW). O-ring clamps were used to lock the optical fibers in the catheters. Each fiber must be carefully pushed to the bottom of the FIC in order to have the illuminating diffusor perfectly positioned into the prostate. To avoid any displacement of the fibers in the prostate, the set of the optical fibers was carefully stuck to the transparent drape with steri-strips. The positions of these fibers defined a precise targeted treatment area that resulted in a sharp controlled necrosis volume according to the treatment guidance. With the optimal treatment conditions: each centimeter of fiber induced 0.8–1 cm^3^ of necrosis with more than 90 % of necrosis of the targeted volume [1]. At the end of this stage of the procedure, the surgeon had to verify that the Light Density Index (LDI) was above one fulfilling an additional optimal condition further to the hemi ablation approach [2]. Before the infusion of the photosensitizer, a safety light test is performed putting a light detection probe in the lumen of the rectum anteriorly to the endocavity balloon. After illuminating all the optical fibers placed into the prostate (“test mode” in the laser generator screen), the rectal light probe was moved in and out until reaching the highest level of light intensity with a maximum allowed of 13 mW/cm^2^. Above this threshold, the first measure taken was to deflate the balloon in order to increase the distance between the posterior part of the prostate and the anterior rectal wall. If it was insufficient, the endorectal probe was moved posteriorly by using the *Z*-axis of the brachystepper in order to obtain an acceptable light intensity level into the anterior rectal wall. Once the adequate light level was obtained, the US probe should not be moved anymore in order to keep the light monitor in place.

Once all FICs and optical fibers were in position, the light of the room was dimmed and the patient was entirely protected from light exposure and wear goggles. The infusion of TOOKAD^®^ Soluble was then initiated through opaque syringe and line.

**Fig. 10:**
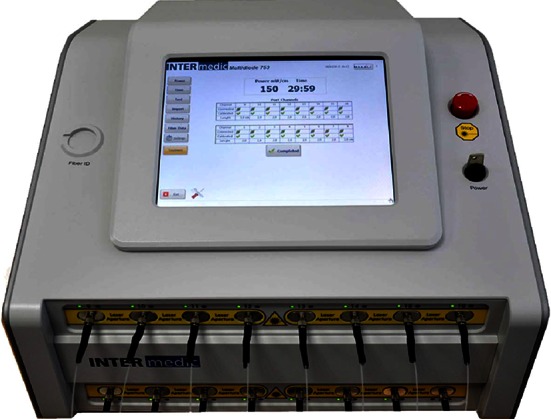
Laser generator

Patient received a 10-min single intravenous administration of 4 mg/kg TOOKAD^®^ Soluble. The activation of the TOOKAD^®^ Soluble was achieved by continuous illumination of the prostate gland through the optical fibers with a 753-nm laser light at a power of 150 mW/cm and light energy of 200 J/cm delivered by a multichannel diode laser (Fig. 10). The illumination started immediately after the end of the injection during 22 min and 15 s in order to coincide with the peak serum concentration of TOOKAD^®^ Soluble [5, 6].

**Fig. 11:**
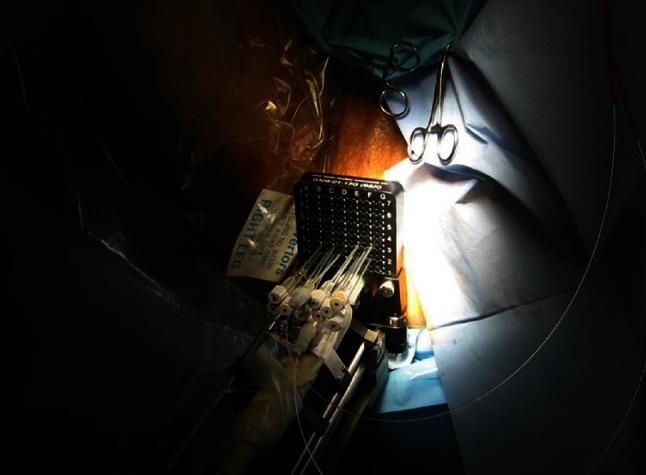
VTP preoperative view

The total duration of the whole procedure lasted between 1.5 and 2 h (including anesthesia, fiber placement and illumination) and depended on the volume of the targeted area and the number of optical fibers to be placed (Fig. 11). At the end of the illumination, the laser was turned off and the FICs and optical fibers were withdrawn at once from the prostate. The indwelling catheter was connected to a urinary bag. It was mandatory keeping the patient completely covered with a sheet during the removal of the sterile drape and during his transfer from the operating table to his bed. In order to ease the communication and to avoid any stress, a bed cradle kept the sheet at distance from the face of the patient until his face could be uncovered.

## Postoperative management

The patient was kept under medical surveillance under dimmed light for at least 6 h and may be discharged on the day of the procedure as a day-case surgery. The patient was informed that he had to avoid direct exposure to sunlight during 48 h after the injection. Dimmed light was recommended the day of the procedure. The urinary catheter could be removed 3–4 h after the procedure or on the following day. An alpha-blocker was prescribed because of the increased risk of LUTS during the first month after the procedure.

## Conclusion

Vascular-targeted photodynamic therapy with TOOKAD^®^ Soluble (WST11) is a standardized procedure for localized prostate cancer treatment. It is relatively simple, easy to learn and easy to perform. It is minimally invasive and requires a short anesthesia (approximatively 2 h), therefor it can be done in ambulatory conditions. The evolution of the technical procedure has been the result hundreds of cases in various environments such as public or private sectors, university or general hospitals. The shared experience on various continents with different medical cultures of the surgeons involved in the studies improved the technique in a way to adapt itself to a vast majority of surgical centers with an optimal safety and efficacy of the treatment.
